# Bibliographical

**Published:** 1899-04

**Authors:** 


### EDITORIAL.

### Bibliographical.

A Manual of Comparative Dental Anatomy for Dental Students. Prepared by request of the National Association of Dental Faculties and adopted as a text-book for colleges, August 27th, 1898. By Alton Howard Thompson, D.D.S., Topeka, Kansas, Professor of Dental Anatomy, Human and Comparative, in Kansas City Dental College, Kansas City, Mo.

This work issued by the S. S. White Dental Manufacturing Co., of Philadelphia, will be received by the profession with much satisfaction, especially by all those engaged and interested in dental teaching. Such a work has been for a number of years greatly needed in our schools, and it has been known by a good many, for three or four years at least, that Dr. Thompson had such a work in preparation. Some two years ago circumstances arose which it was feared might interrupt, if not prevent, the completion of the work.

The author in the preface says the sifting out of the special matter from the mass of material, bearing on the subject in the literature of zoology, has been the work of years, and represents the gleanings from many fields. Its matter and methods have been slowly evolved through the needs of the work of teaching this branch in the class-room, and it is hoped that it will be useful to teachers of this branch. The best place for this branch in the curriculum will be as a preliminary study in the course of dental anatomy preceding and leading up to human dental anatomy. It begins with the lowest forms of life and leads up to the highest in regular gradation—taking the teeth seriatim from the lowest types, and showing their progressive evolution from simple to complex forms. A list of questions has been appended to each chapter which will be an aid to the teacher as well as to the student in quiz class.

This little work presents the salient points of the subject of which it treats in a very systematic and thorough manner. In the cursory examination which we have been able to make, we find nothing to correct or criticise. The arrangement is very good indeed, and the presentation of the subject will very thoroughly serve the purpose for which it was intended. In treating the subject, the author begins with the most simple form of dental apparatus, going on step by step to the higher, until the most complex dental apparatus is reached—that of man—presenting the more striking points of each variety from the beginning to the end. On page 22 will be found the nomenclature of Comparative Dental Anatomy, which is here presented in a very concise form.

In looking over the work the wonder forces itself upon the mind that so much valuable information on this subject can be condensed into one hundred and seventy-six pages. Very few men in our profession are so well qualified for preparing such a work as Dr. Thompson, having been engaged in teaching this subject for a number of years, and drawing his material from every available source. The work can be procured from the publisher, at the dental depots, or from regular book-sellers.

